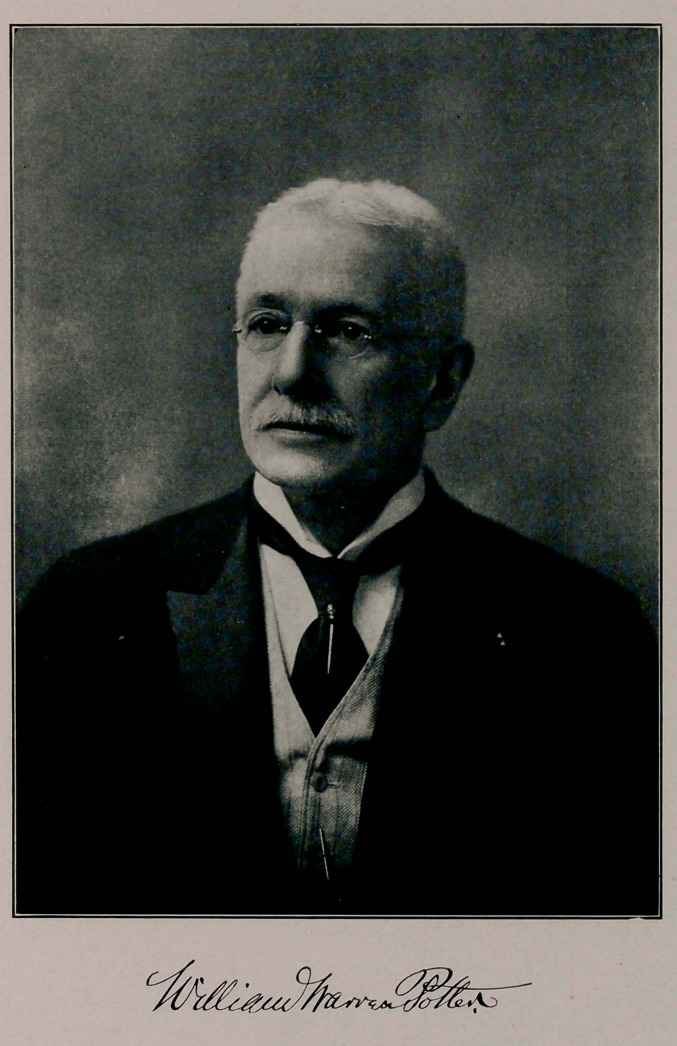# William Warren Potter

**Published:** 1911-04

**Authors:** L. S. McMurtry


### BUFFALO MEDICAL JOURNAL

**A Monthly Review of Medicine and Surgery**

*EDITOR*

WILLIAM WARREN POTTER, M. D.

All communications, whether of a literary or business nature, books for review and exchanges, should be addressed to the editor, 238 DELAWARE AVENUE, BUFFALO, N.Y.

VOL. LXVI. APRIL, 1911. No. 9

### William Warren Potter

ON Tuesday morning, March 14, the Editor of the BUFFALO MEDICAL JOURNAL died in this city after a month's severe illness. Until he was confined to his bed he gave to every department of the JOURNAL the most unremitting attention, with such editorial skill and ability that won and maintained for it the confidence of the profession. He made it a constant influence for good, diffusing the best of advancing knowledge and constantly holding aloft the standard of professional honor and achievement. This work was with him for the most part altruistic, and was prompted by his devotion to medicine and the medical profession of his county and state. It is impossible to properly estimate the value of a service so long and faithfully discharged, with an influence so far-reaching in its results.

Dr. Potter was signally honored throughout his long and active professional career both by the members of the profession and by distinguished citizens of the state of New York. Very soon after his admission to the profession, he enlisted as a surgeon in the Federal army and served throughout the great civil war. He was actively engaged with the troops in the field, enduring the hardships of camp and battle-field. He was promoted to Division Surgeon, and brevetted by the President of the United States, for faithful and meritorious service, lieutenant colonel of United States volunteers, and by the governor of New York state, for like reasons, lieutenant colonel of New York volunteers.

He was a member of the American Medical Association, and in 1890 was elected chairman of the section of Diseases of

William Warren Potter

Women. In 1891 he was elected president of the Medical Society of the State of New York. In 1893 he was president of the Medical Society of the County of Erie, and during the same year he organised the Section of Gynecology and Abdominal Surgery of the Pan-American Medical Congress. For the last fourteen years he was president of the Board of Medical Examiners of the State of New York, and discharged the exacting duties of this office with ability and the most conscientious care.

Dr. Potter was a liberal contributor to medical literature upon practical subjects, specialising in gynecology and obstetrics, during the years he was actively engaged in hospital and private pracice. During the eventful years back in the eighties when gynecology was passing through a period of active development into its present high position of finished achievement, he made numerous valuable contributions to this department of surgery. Perhaps the most far-reaching and potential of his public professional services were rendered through his labors as permanent secretary of the American Association of Obstetricians and Gynecologists. He was one of the founders of this national organisation, and has been its only secretary and editor of the Transactions throughout the twenty-two years of its existence. He possessed rare skill for the difficult work of such an office. Accurate and methodical, with a capacity for details most unusual, he was recognised as *facile princeps* in discharging the duties of this office. He was quick to recognise true worth, and brought many men into prominence by affording the opportunity to develop ability and skill who otherwise might have remained in obscurity. The literature of gynecology and abdominal surgery has been enriched, and the practice of this special department of surgery has been materially advanced by this organisation of which he was from the beginning the moving spirit.

Any notice of Dr. Potter would be incomplete without some allusion to those delightful social qualities which made him a favorite wherever he was known. In bearing, in manners and dress, he was the typical refined and courtly gentleman. He was an honorable man in all his professional and private relations, and to those who enjoyed his confidence he was a true and loyal friend. He occupied a prominent and responsible place in the profession through a long period of years, and discharged his trust with honor and good faith. And now he rests.

L. S. McMurtry.

## Figures and Tables

**Figure f1:**